# The impact of the COVID-19 pandemic on stress, mental health and coping behavior in German University students – a longitudinal study before and after the onset of the pandemic

**DOI:** 10.1186/s12889-021-11295-6

**Published:** 2021-07-13

**Authors:** Edgar Voltmer, Susen Köslich-Strumann, Anna Walther, Mahmoud Kasem, Katrin Obst, Thomas Kötter

**Affiliations:** 1 Institute for Social Medicine and Epidemiology, University of Lübeck, Ratzeburger Allee 160, 23562 Lübeck, Germany; 2 Institute of Family Medicine, University Medical Centre Schleswig-Holstein, Ratzeburger Allee 160, 23562 Lübeck, Germany

**Keywords:** COVID-19, Students, Mental health, Preventive measures, Behavior patterns

## Abstract

**Background:**

The COVID-19 pandemic has led to massive restrictions in public and private lives, including a shut-down of face-to-face teaching at universities in Germany. We aimed to examine the impact of these changes on perceived stress, mental health and (study-)related health behavior of students in a longitudinal study.

**Methods:**

For two timepoints – the year before the COVID-19 pandemic (2019, *n* = 1377) and the year during the COVID-19 pandemic (2020, *n* = 1867) – we surveyed students of all faculties at one German university for perceptions and preventive behavior regarding the COVID-19 pandemic using standard instruments for stress, anxiety, depression, and behavior and experience patterns.

**Results:**

About 90% of students (*n* = 1633) in 2020 did not have a known contact infected with SARS-CoV-2, while 180 (9.8%) did have one. Only 10 respondents (0.5%) reported an infection with SARS-CoV-2. Wearing masks and washing hands more often were practiced by ≥80% of students. Taking more care about cleanliness (51.8%) and using disinfectants (39.2%) were practiced much less. A higher percentage of female compared with male students and medical/health science students compared with science, technology, engineering, and mathematics students engaged more frequently in specific or nonspecific preventive measures. More than three quarters (77.1%) of all students rated their general health as (very) good. There were no significant differences in general health, stress, and depression between 2019 and 2020 in the students who responded at both timepoints. The distribution of behavior and experience patterns for this group showed a slight but significant difference from 2019 to 2020, namely decreasing proportions of students with a healthy pattern and a risk pattern for overexertion. Students with different behavior and experience patterns showed marked differences in perceptions and reaction to the COVID-19 pandemic as well as psychosocial stress and symptoms, with higher scores for mental health symptoms and lower scores in preventive behavior regarding risk patterns.

**Conclusion:**

Despite massive alterations to students’ lives in 2020, there were only moderate consequences for mental health compared with 2019 in the total student group of this German university. However, identifying students at risk would offer opportunities to foster mental health in relevant subgroups.

**Supplementary Information:**

The online version contains supplementary material available at 10.1186/s12889-021-11295-6.

## Background

For the first time in recent history, due to the COVID-19 pandemic public life was massively restricted, including a complete interdiction of face-to-face learning/teaching at public schools and universities. Within a short period of time, teaching and learning had to be adjusted to mostly digital and online formats [1, 2]. Academic life, as well as the private life of students have been affected massively by the impact of regulations for social contacts and hygiene [3, 4].

Students’ perceptions and reactions to such unpredictable and threatening life events have been reported from different countries. In a study from China, a quarter of students were afraid because of the COVID-19 outbreak [5]. In an online survey of Turkish students about their emotions regarding COVID-19, 38% responded as being worried [6]. Studies from within the European Union have reported an increase in stress and anxiety for about 60% of students in France, Spain, and Poland [7–9] due to the COVID-19 pandemic. From universities in different federal states of Germany, about 40–60% of students reported increased mental stress, feelings of loneliness, and fear of the future [10–12]. It has to be noted though that about 17% of Bavarian students indicated that they have experienced less mental stress through the COVID-19 pandemic [10]. Most of these recent studies are cross-sectional and have estimated the difference to the time before the pandemic by using ex-post items. Real longitudinal studies that compare the impact of the COVID-19 pandemic in the same population before and during the pandemic are rare. From a longitudinal study in Swiss undergraduate students, there was an increase in depression, anxiety, stress, and loneliness from within-person comparisons. By contrast, the authors could not identify a significant difference in these parameters in between-cohort comparisons [13]. In Italian students, the initial increase in depressive symptoms during lockdown disappeared after the lockdown ended; only 6% were at risk for the development of severe depressive symptoms [14].

Based on past virus outbreaks, the World Health Organization (WHO) and the Centers for Disease Control and Prevention (CDC) recommended preventive measures for the general population, like wearing a mask or washing hands more often, as well as more general recommendations for a healthy lifestyle (e.g., exercise, nutrition, sleep). These recommendations were adopted for the actual COVID-19 pandemic worldwide [15]. However, adherence to the recommendations has shown regional and group-related differences [2, 6, 16]. Regarding sleep, as an example of a healthy lifestyle behavior, reviews in students have reported that sleep problems have been highly prevalent with an increasing tendency [17, 18]. Much smaller proportions of the general population and medical personnel have reported such problems. However, sleep problems have been frequently seen in COVID-19 patients [19].

Social support is an important factor in the prevention of mental illness and the promotion of health [20]. In a study of U.S. medical students, resilient students who did not have symptoms of burnout at different timepoints of the course of study reported greater social support than their vulnerable fellow students with burnout symptoms [21]. It was observed that students returned home during the COVID-19 pandemic because their social network on campus was no longer available [22]. A smaller proportion of students who relocated to places with their parents before confinement, reported stress during confinement (51%) compared with students who did not relocate (72%) [7]. Living with parents and social support of relatives were also protective factors against anxiety [5, 8].

There is increasing evidence that different patterns of students’ behavior and experience could be identified. For example, based on a salutogenic approach, Schaarschmidt et al. [23] described a healthy pattern, an unambitious pattern, and two risk patterns of overexertion and burnout. These patterns reflect the experience of study-related stress and typical coping behaviors (Work/Study-Related Behavior and Experience Pattern [Arbeitsbezogenes Verhaltens- und Erlebensmuster, AVEM], see also the Methods section). Students with different patterns also differ in factors like perception of stress, physical and mental health, anxiety, and depression [24].

The perception of aspects related to the COVID-19 pandemic might not only differ in patterns, but also in gender. Sixty-one percent of female compared with 49% of male students reported a (very) negative impact on their mental health [12]. Other studies have confirmed a higher vulnerability of female students regarding stress and increased mental health symptoms due to the COVID-19 pandemic [10, 13, 25].

The aim of this study was to examine students’ perceptions of risks and preventive behavior regarding the COVID-19 pandemic. We compared the occurrence of stress and mental health symptoms at the time of the COVID-19 pandemic (2020) with the year before (2019), which was unaffected by this disease, to determine whether these factors predict anxiety and preventive behavior related to the COVID-19 pandemic in 2020. We also analyzed the differences of the four behavior and experience patterns (AVEM) in these parameters and the influence of gender and social support.

## Methods

We compared two timepoints – the year before (2019) and the year during (2020) the COVID-19 pandemic – in an ongoing prospective, longitudinal, observational study including all students at one German university (University of Lübeck, UzL) [26]. The follow-up surveys were taken online in June during the respective summer semesters. A €5 book or food voucher per completed questionnaire was used as an incentive for participants. For the identification of datasets in the longitudinal analyses, the participants were asked to generate a personal identification code and/or provide their matriculation number. Cross sectional analysis was conducted for all students who participated. Sub-analyses were performed for separate groups of students according to field of study: medicine (MED); health sciences (HSC); and science, technology, engineering and mathematics (STEM). The following instruments and items were included in the survey (see also the supplementary file).

### Selected items on COVID-19 pandemic perceptions and measures (2020 only)

For COVID-19 pandemic-related perceptions and behavior, we used a set of items that we developed or that we retrieved from polls and the literature. These were: “Did you have contact to a person infected with coronavirus?” (yes/no). “Did you yourself suffer a coronavirus infection?” (yes, no, not known). If no or not known, the next two items derived from polls of the general population were administered: “How much are you afraid about contagion with the coronavirus?,” rated on a 4-point Likert scale (very afraid, afraid, slightly afraid, almost not afraid) [27]. “Suppose you were infected by the coronavirus, how seriously do you think COVID-19 would affect your health” (Extremely, very seriously, seriously, moderate, not much) [28]. In addition, a self-developed item was administered: “Which areas of your life are severely affected by the COVID-19 pandemic?,” with the following response options: study, economic, and intangible (emotion, meaning) existence, rated on a 5-point Likert scale from 1 – not at all to 5 – very much. Finally, eight preventive health behaviors recommended by WHO and CDC were included rated on a 5-point Likert scale from 1 – never to 5 – always [15]: wearing a mask, washing hands more often, taking more care about cleanliness, using disinfectants, eating a balanced diet, exercising regularly, taking an herbal supplement, making sure they got sufficient sleep, and complemented by a self-generated item: contacting friends and family.

The following instruments were administered at both timepoints (2019/2020).

### General health

General Health was surveyed with one item “How would you describe your general health?,” rated on a 5-point Likert scale from 1 – very good to 5 – very poor [29].

### Perceived stress scale (PSS)

The PSS comprises 10 items rated on a 5-point Likert-scale from 1 – never to 5 – very often [30]. Of the original 14-item scale, four items were dropped by the authors of the instrument because of low factor loadings. Furthermore, the 10-item version has been validated in a German sample and norm values for the German population are available [31]. It measures the perception of respondents about their lives being (un-)predictable, (un-)controllable, and overloaded (e.g.,” How often have you felt that you could not control the important things in your life?”). Cronbach’s alphas in the present study were 0.87 (2019) and 0.89 (2020).

### Brief symptom inventory (BSI-18)

The BSI-18 is the latest and shortest of the multidimensional versions of the Symptom-Checklist 90-R (SCL 90-R). We used the subscales for depression and anxiety with six items each rated on a 5-point Likert Scale from 1 – not at all to 5 – very much [32]. Cronbach’s alphas for anxiety in the present study were 0.81 (2019) and 0.81 (2020), and for depression they were 0.86 (2019) and 0.84 (2020).

### Work-related behavior and experience pattern (AVEM)

We used the 44-item short form of the standard instrument: “Work-Related Behavior and Experience Pattern [Arbeitsbezogenes Verhaltens- und Erlebensmuster, AVEM] [23] in a student-adapted version. The AVEM allows one to examine personal experiences with study related stress and typical coping behaviors. On the basis of 11 health-relevant dimensions from the domains of professional ambition, resistance toward stress, and emotional wellbeing (in the context of work/study), four health-relevant behavior and experience patterns could be identified.

#### Pattern G (healthy)

Students with pattern G are characterized by a good balance between study-related ambition and the ability to cope with stress. They are satisfied with study and life and the experience of social support.

#### Pattern S (unambitious)

In contrast to pattern G, this pattern is characterized by lower scores in the dimensions of study-related commitment while the ability to cope with stress and emotional well-being remain positive. There is a certain ambivalence to this pattern. The lack of study-related ambition could either be a relaxed attitude whereby one does not take study very seriously and is interested and finds satisfaction in activities outside the course of study. However, it could also be an early sign of frustration and loss of motivation that could lead to later burnout.

The next two patterns are deliberately termed risk patterns because it has been repeatedly shown that they correlated with poor health and illness [33, 34].

#### Risk pattern a (overexertion)

Students with this pattern are characterized by overcommitment and a low ability to cope with stress. Their emotional wellbeing is impaired. The label was chosen to indicate the resemblance to type A behavior, described by Friedman and Rosenmann as a risk factor for coronary artery disease and myocardial infarction [35].

#### Risk pattern B (burnout)

In students with this pattern, we find lower study-related ambition, poor ability to cope with stress, and impaired emotional well-being.

The validation of the instrument showed moderate to good correlations to related scales (e.g., the Freiburg Personality Inventory (FPI), the Maslach Burnout Inventory (MBI), and the Big-Five Adjective List) [36, 37].

For demographics, age, sex, and study group (MED,STEM, HSC) were included.

### Statistical analysis

The statistical analysis was conducted with SPSS (version 22.0, SPSS Inc., Chicago, IL). We report univariate statistics as means and standard deviations for continuous variables and percentages for categorical variables. For categorical variables, data were analyzed using chi-squared-tests for cross-sectional analysis and the McNemar-Bowker test for longitudinal analysis. For continuous variables, data were analyzed using two tailed t-test. Differences between AVEM patterns were analyzed with analysis of variance (ANOVA). The influence of the independent variables on anxiety and the preventive measure of washing hands were analyzed with linear regression models with cut-off scores of *p* < 0.05 for inclusion. A correlation matrix of all variables included in the survey is provided as a supplementary file.

## Results

In the 2018/2019 study year a total of 4774 students (female 2810, male 1964) were enrolled at Lübeck university. In the 2019/2020 study year, there were a total of 5477 students (female 3215, male 2262). In 2019, *n* = 1377 (28.8%) students responded to the surveys, and in 2020, *n* = 1867 (34.1%) responded. Three quarters of the respondents were female, about 50% from STEM, about 40% from MED and almost 10% from HSC (s. Table 1). Compared with all students enrolled at UzL, MED and HSC students and female students were more prone to participate.

**Table 1:** Sociodemographic description of study participants

	2019^a^		2020^a^		Longitudinal 2019/2020^b^	
	n	%	n	%	n	%
**Sex**						
Male	325	24.4	413	24.2	175	20.9
Female	1000	75.2	1286	75.2	659	78.7
Divers	5	0.4	10	0.6	3	0.4
**Age M (SD)**	1331	23.5 (3.4)	1709	23.8 (3.5)	24.1 (3.3)	
**Study group**						
MED^c^	595	43,4	745	41.1	397	45.5
STEM	653	47.6	913	50.4	417	47.8
HSC	123	9.0	153	8.4	58	6.7

^a^cross-sectional analysis for 2019 and 2020

^b^longitudinal analysis (all participants who responded in 2019 and 2020)

^c^HSC: health sciences; MED: medicine; STEM: science, technology, engineering, and mathematics

### Perceptions and preventive behavior regarding the COVID-19 pandemic

More than three quarters (77.1%) of all students rated their general health as (very) good. About 90% of students (*n* = 1633) in 2020 did not have contact with a person infected with SARS-CoV-2 while 180 (9.8%) did. Only 10 respondents (0.5%) reported an infection with SARS-CoV-2. Of those who did not report an infection (64.2%) or did not know about it (35.3%), only 8.3% reported to be (very) afraid about being infected, and for 45.3% this was only a minor concern. About 56% of students felt that an infection with SARS-CoV-2 would affect their health only moderately, 27.5% even less, and only about 16% (very) much.

**Fig. 1:**
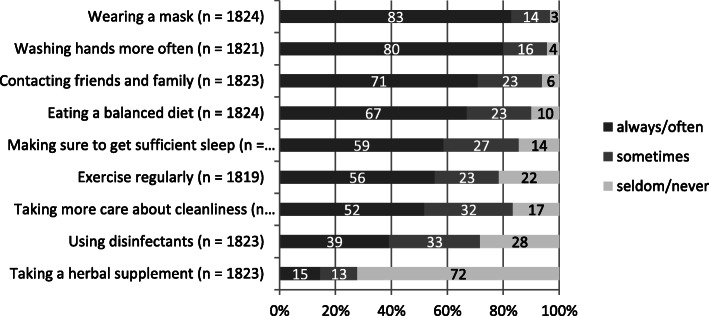
Frequency of preventive measures in all students (2020)

The use of different preventive measures showed varying degrees of intensity depending on the measure (Fig. 1). Wearing masks and washing hands more often were practiced by ≥80% of students always/often. Taking more care about cleanliness (51.8%) and using disinfectants (39.2%) were practiced to a much lesser degree.

There were differences between gender and field of study (Table 2). Women showed a higher implementation of measures than men. This is particularly evident in the aspects of eating a balanced diet (70.1% vs. 59.3% always/often), getting enough sleep (61.9% vs. 48.3%), and contacting friends and family (75.6% vs. 56.6%; *p* < 0.01). MED and HSC students more often wore a mask (MED 84.4%, STEM 81.1%, HSC 86.9% always/often) and paid more attention to contacting friends and family than STEM students (MED 77.4%, STEM 65.0%, HSC 73.9%). In addition, MED students were physically more active (MED 64.0%, STEM 50.1%, HSC 49.0%). Almost all students (94%) (strongly) agreed that the preventive measures for the limitation of the pandemic by the government were effective.

**Table 2:** Frequency of preventive measures in all students by sex and study group (2020)

	Total	Always/often		Sometimes		Seldom/never		p^a^
	n	n	%	n	%	n	%	
**Wearing a mask**	1824	1513	82.9	251	13.8	60	3.3	
Female	1285	1087	84.6	160	12.5	38	3.0	0.037
Male	413	327	79.2	69	16.7	17	4.1	
MED	744	628	84.4	96	12.9	20	2.7	0.190
STEM	909	737	81.1	135	14.9	37	4.1	
HSC	153	133	86.9	17	11.1	3	2.0	
**Washing hands more often**	1821	1457	80.0	285	15.7	79	4.3	
Female	1285	1043	81.2	197	15.3	45	3.5	0.031
Male	410	313	76.3	72	17.6	25	6.1	
MED	744	574	77.2	128	17.2	42	5.6	0.067
STEM	906	737	81.3	136	15.0	33	3.6	
HSC	153	130	85.0	19	12.4	4	2.6	
**Contacting friends and family**	1823	1292	70.9	419	23.0	112	6.1	
Female	1285	971	75.6	261	20.3	53	4.1	0.000
Male	412	233	56.6	130	31.6	49	11.9	
MED	743	575	77.4	138	18.6	30	4.0	0.000
STEM	909	591	65.0	243	26.7	75	8.3	
HSC	153	113	73.9	34	22.2	6	3.9	
**Eating a balanced diet**	1824	1221	66.9	421	23.1	182	10.0	
Female	1285	901	70.1	281	21.9	103	8.0	0.000
Male	413	245	59.3	107	25.9	61	14.8	
MED	744	536	72.0	161	21.6	47	6.3	0.000
STEM	910	576	63.3	215	23.6	119	13.1	
HSC	152	97	63.8	41	27.0	14	9.2	
**Make sure to get enough sleep**	1823	1069	58.6	491	26.9	263	14.4	
Female	1285	795	61.9	329	25.6	161	12.5	0.000
Male	412	199	48.3	133	32.3	80	19.4	
MED	742	456	61.5	190	25.6	96	12.9	0.143
STEM	910	511	56.2	253	27.8	146	16.0	
HSC	153	93	60.8	43	28.1	17	11.1	
**Exercise regularly**	1819	1010	55.5	416	22.9	393	21.6	
Female	1282	729	56.9	297	23.2	256	20.0	0.059
Male	412	218	52.9	89	21.6	105	25.5	
MED	741	474	64.0	165	22.3	102	13.8	0.000
STEM	907	454	50.1	207	22.8	246	27.1	
HSC	153	75	49.0	39	25.5	39	25.5	
**Taking more care about cleanliness**	1822	944	51.8	576	31.6	302	16.6	
Female	1284	685	53.3	401	31.2	198	15.4	0.034
Male	412	193	46.8	137	33.3	82	19.9	
MED	740	384	51.9	233	31.5	123	16.6	0.863
STEM	911	465	51.0	295	32.4	151	16.6	
HSC	153	85	55.6	43	28.1	25	16.3	
**Using disinfectants**	1823	715	39.2	592	32.5	516	28.3	
Female	1285	506	39.4	431	33.5	348	27.1	0.030
Male	412	159	38.6	116	28.2	137	33.3	
MED	744	290	39.0	257	34.5	197	26.5	0.000
STEM	908	336	37.0	281	30.9	291	32.0	
HSC	153	80	52.3	49	32.0	24	15.7	
**Taking herbal supplement**	1823	267	14.6	240	13.2	1316	72.2	
Female	1285	210	16.3	172	13.4	903	70.3	0.004
Male	412	44	10.7	45	10.9	323	78.4	
MED	743	104	14.0	89	12.0	550	74.0	0.182
STEM	909	146	16.1	128	14.1	635	69.9	
HSC	153	16	10.5	19	12.4	118	77.1	

^a^Pearson’s chi-squared test; *p* values are for the differences between female and male, as well as between the study subgroups of medicine (MED); science, technology, engineering and, mathematics (STEM); and health sciences (HSC) in the use of preventive measures

### Impact of the COVID-19 pandemic on study, economic and intangible existence and mental health

Eighty-five percent of the students stated that the COVID-19 pandemic had had an influence on their studies. Women stated this significantly more often than their fellow male students (86.9% vs. 81.4%, *p* < 0.01). Almost a quarter of the students (23.9%) said that the COVID-19 pandemic had had an influence on their economic existence. STEM students stated this significantly more frequently (30.4%) than the other students (MED 18.1% HSC 22.2%). Almost 70% of the students indicated that the situation had had an influence on their intangible existence (emotion, sense of life). This was significantly more often the case for female students (69.2% vs. 62.7%) and STEM students (71.6% STEM, MED 64.2%, HSC 66.4%; Table 3).

**Table 3:** Perceptions of all students about the impact of the COVID-19 pandemic on their course of study and their economic and intangible existence (2020)

Impact of COVID-19 pandemic on…						
		Strongly agree/agree/ undecided		Disagree/highly disagree		p^a^
	Total n	n	%	n	%	
**study**	1821	1549	85.1	272	14.9	
Female	1282	1114	86.9	168	13.1	0.005
Male	413	336	81.4	77	18.6	
MED	744	647	87.0	97	13.0	0.024
STEM	907	753	83.0	154	17.0	
HSC	152	136	89.5	16	10.5	
**economic existence**	1822	449	24.6	1373	75.4	
Female	1283	306	23.9	977	76.1	0.802
Male	413	101	24.5	312	75.5	
MED	741	134	18.1	607	81.9	0.000
STEM	910	277	30.4	633	69.6	
HSC	153	34	22.2	119	77.8	
**intangible existence**	1823	1244	68.2	579	31.8	
Female	1284	889	69.2	395	30.8	0.014
Male	413	259	62.7	154	37.3	
MED	743	477	64.2	266	35.8	0.005
STEM	910	652	71.6	258	28.4	
HSC	152	101	66.4	51	33.6	

^a^Pearson’s chi-squared test; *p* values are for the differences between female and male, as well as between the study subgroups of medicine (MED); science, technology, engineering, and mathematics (STEM); and health sciences (HSC) in the use of preventive measures

Students who rated that the COVID-19 pandemic had had an impact on their study situation showed significantly higher values in their stress levels than students who stated there had been no relevant impact of the COVID-19 pandemic on their study situation (*p* < 0.01). The same was true for students who quoted that the COVID-19 pandemic had had a relevant impact on their economic and intangible existence who also showed significantly higher values in anxiety and depression (*p* < 0.01; Table 4).

**Table 4:** Differences in students with or without a perceived impact of the COVID-19 pandemic on stress, depression, and anxiety (2020)

Impact of COVID-19 pandemic on…	Group	n	Mean	SD	p
**study**					
Stress	Impact^a^	1537	28.7	6.9	0.000
	No impact^b^	268	25.9	7.1	
Depression	Impact	1329	5.0	4.9	0.144
	No impact	181	4.4	5.1	
Anxiety	Impact	1328	4.5	4.3	0.168
	No impact	181	4.0	4.1	
**economic existence**					
Stress	Impact	445	30.8	6.6	0.000
	No impact	1360	27.5	6.9	
Depression	Impact	384	6.3	5.5	0.000
	No impact	1128	4.5	4.6	
Anxiety	Impact	383	5.2	4.8	0.000
	No impact	1128	4.1	4.1	
**intangible existence**					
Stress	Impact	1231	29.9	6.6	0.000
	No impact	576	24.9	6.5	
Depression	Impact	1042	5.7	5.0	0.000
	No impact	470	3.2	4.1	
Anxiety	Impact	1041	4.9	4.5	0.000
	No impact	470	3.4	3.7	

^a^Impact response options: strongly agree, agree, or undecided

^b^No impact response options: disagree or highly disagree

For the longitudinal analyses, 890 students who responded at both time points were included (female *n* = 659 (78.7%); MED *n* = 397 (45.5%), STEM *n* = 417 (47.8%), HSC *n* = 58 (6.7%); Table 1).

There was no significant difference between the scores of general health, stress, and depression in 2019 and 2020 in those students who responded at both timepoints. Paired sample t-tests showed a significant decrease in social support and anxiety between 2019 and 2020, but the differences in mean values were small (Table 5). However, it might be suggested that paired sample t-tests would not consider sufficiently the correlational nature of longitudinal data. Therefore, we also calculated fixed effects models for the variables mentioned in Table 5. These models did not reveal any significant differences for the variables between 2019 and 2020.

**Table 5:** Differences in general health, social support, stress, anxiety, and depression before (2019) and during (2020) the COVID-19 pandemic (participants who answered at both timepoints)

Outcome		n	Mean	SD	p
General health	2020	886	2.1	0.8	0.401
	2019		2.1	0.8	
Social support	2020	851	16.8	2.8	0.008
	2019		17.0	2.6	
Stress	2020	695	27.5	8.0	0.547
	2019		27.4	7.7	
Anxiety	2020	587	4.1	4.1	0.025
	2019		4.5	4.4	
Depression	2020	588	4.5	4.8	0.089
	2019		4.8	5.0	

**Fig. 2:**
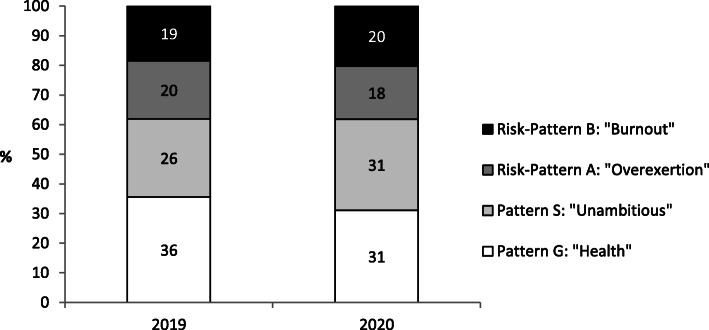
Behavior and experience patterns of all students (*n* = 848) who answered at both timepoints (2019 and 2020)

There was a slight but significant difference in the distribution of behavior and experience patterns (AVEM) of all students from 2019 to 2020 (McNemar-Bowker = 20.7, df = 6, *p* < 0.01). The proportion of students with the healthy pattern fell by 5%, and correspondingly the proportion of those with an unambitious pattern rose by the same amount. The risk pattern for overexertion fell by 2% (Fig. 2).

Students with different behavior and experience patterns (AVEM) showed marked differences in perceptions and reactions to the COVID-19 pandemic as well as psychosocial stress and symptoms. For fear of infection and the impact of the COVID-19 pandemic on study, economic, and intangible existence as well as the more specific protective measures (e.g., wearing a mask, washing hands more often), the unambitious pattern S and the overambitious risk pattern A marked the extremes (with the lowest scores in the preventive measures for those students with the unambitious pattern S and the highest for the risk pattern A for overexertion) while the healthy pattern G and the risk pattern B were more intermediate. By contrast, for nutrition, exercise, and social support from friends and family, those students with the healthy pattern G had the highest scores and those with the risk pattern B had the lowest. For stress, anxiety, and depression, it was exactly reverse (Table 6).

**Table 6:** Differences of behavior and experience patterns (AVEM) in study variables

	Pattern	n	Mean	SD	Welch’s ANOVA posthoc^a^ *p* < 0.05
Afraid of infection	G	544	1.6	0.6	F(3, 890.8) = 20.7, *p* < 0.01 GS^b^, GA, GB, SA, SB
	S	482	1.5	0.6	
	A	352	1.8	0.7	
	B	367	1.7	0.7	
Impact on study	G	544	3.7	1.2	F(3, 926.1) = 7.1, *p* < 0.01 SA, SB
	S	482	3.5	1.1	
	A	349	3.9	1.0	
	B	367	3.8	1.1	
Impact on economic existence	G	544	1.9	1.0	F(3, 889.8) = 16.3, *p* < 0.01 GA, GB, SA, SB
	S	479	1.8	0.9	
	A	351	2.2	1.1	
	B	368	2.1	1.1	
Impact on intangible existence	G	544	2.8	1.1	F(3, 911.4) = 31,8, *p* < 0.01 GA, GB, SA, SB
	S	481	2.8	1.1	
	A	352	3.3	1.1	
	B	367	3.3	1.1	
Wearing a mask	G	544	4.1	0.8	F(3, 915.3) = 7.5, *p* < 0.01 GS, SA
	S	482	3.9	0.7	
	A	351	4.2	0.7	
	B	368	4.1	0.7	
Washing hands more often	G	544	4.2	0.8	F(3,905.5) = 13.4, *p* < 0.01 GS, SA, AB
	S	482	3.9	0.9	
	A	351	4.2	0.8	
	B	365	4.0	0.9	
Taking more care about cleanliness	G	542	3.6	1.0	F(3, 911.1) = 27.3, *p* < 0.01 GS, GA, GB, SA, SB, AB
	S	482	3.2	1.0	
	A	351	3.8	1.0	
	B	368	3.4	1.0	
Using disinfectants	G	544	3.2	1.1	F(3, 906,0) =13.2, *p* < 0.01 GS, GA, SA, AB
	S	482	2.9	1.0	
	A	352	3.4	1.1	
	B	366	3.1	1.1	
Balanced diet	G	544	4.0	0.9	F(3, 906.8) = 15.7, *p* < 0.01 GS, GA, GB, SB, AB
	S	481	3.8	1.0	
	A	352	3.8	1.0	
	B	368	3.6	1.0	
Regular excercise	G	543	3.8	1.1	F(3, 899.7) = 24.5, *p* < 0.01 GS, GA, GB, SA, SB
	S	481	3.6	1.1	
	A	352	3.4	1.2	
	B	365	3.2	1.2	
Herbal supplements	G	543	1.9	1.2	F(3, 900.1) = 8.2, *p* < 0.01 GA, SA, SB
	S	482	1.8	1.1	
	A	352	2.1	1.3	
	B	367	2.1	1.3	
Sufficient sleep	G	543	3.7	0.9	F(3, 899.3) = 15.1, *p* < 0.01 GA, GB, SA, SB
	S	481	3.7	1.0	
	A	352	3.5	1.0	
	B	368	3.3	1.0	
Friends and family	G	543	4.1	0.8	F(3, 928.5) = 34.1, *p* < 0.01 GS, GA, GB, SA, SB, AB
	S	482	3.9	0.8	
	A	352	3.7	0.8	
	B	367	3.5	0.9	
Stress	G	544	24.9	7.2	F(3, 921.5) = 213.4, *p* < 0.01 GS, GA, GB, SA, SB, AB
	S	481	25.9	6.9	
	A	352	31.3	6.4	
	B	368	33.5	6.0	
Somatic symptoms	G	460	2.3	2.7	F(3, 737.8) = 59.4, *p* < 0.01 GA, GB, SA, SB
	S	388	2.4	2.6	
	A	312	4.6	4.4	
	B	330	5.3	4.7	
Depression	G	460	2.5	2.9	F(3, 733.8) = 153.6, p < 0.01 GS, GA, GB, SA, SB, AB
	S	388	3.3	3.4	
	A	312	6.1	4.7	
	B	330	8.9	5.6	
Anxiety	G	460	2.8	2.8	F(3, 733.5) = 91.5, *p* < 0.01 GA, GB, SA, SB
	S	388	2.9	2.7	
	A	312	6.3	4.8	
	B	330	6.6	5.1	

^a^Games-Howell post-hoc test

^b^Indicates a significant difference between pattern G (healthy) and pattern S (unambitious)

### Predictors of anxiety and washing hands more often

We tested age and sex (model one); stress, anxiety and depression scores from 2019 (model 2); and the fear of a SARS-COV-2 infection, severe health consequences, and perceived consequences on study, economic and intangible existence (model 3) as predictor variables for the dependent variable of anxiety in 2020. We did not include stress, anxiety, and depression scores from 2020 in the model to avoid effects of reverse causality. In the final model (corrected R^2^ = 0.35, F (10, 572) = 32.8, *p* < 0.01) the most important and significant predictors for anxiety in 2020 were anxiety and depression in 2019 followed by sex and consequences for the study and intangible existence (anxiety in 2019 (ß = 0.4, T (572) = 8.1, *p* < 0.01; depression in 2019: ß = 0.2, T (572) = 3.6, *p* < 0.01; sex (β = 0.08, T (572) = 2.4, *p* < 0.05); perception that the COVID-19 pandemic has had an impact on one’s course of study (β = − 0.08, T (572) = − 2.1, *p* < 0.05); and impact on intangible existence (β = 0.1, T (572) = 3.9, *p* < 0.01).

Predictors for washing hands more often were fear of infection (β = 0.2, T (572) = 3.9, *p* < 0.01), fear of consequences for health (β = 0.2, T (572) = 4.1, *p* < 0.01), fear of consequences of the COVID-19 pandemic for economic existence (β = 0.09, T (572) = 2.2, *p* < 0.05), and sex (β = 0.08, T (572 = 2.0, *p* < 0.05); these predictors explained 10.5% of the variance. Stress, anxiety, and depression in 2019 had no significant influence. A separate model on the influence of social support in 2020 on anxiety in 2020 explained less than 1% of the variance.

## Discussion

As one of the rare prospective, longitudinal studies regarding the effects of the COVID-19 pandemic, our results compare general health, perceived stress, mental health symptoms, and study-related behavior and experience patterns (AVEM) of German university students during the COVID-19 pandemic with 1 year before the pandemic. We also report the adherence to COVID-19-specific and general preventive measures and predictors of anxiety and specific preventive measures in 2020.

### Health and mental symptoms

Considering the entire sample, more than three quarters (77%) of students reported good or very good health, and this did not show significant differences to the year before. The proportion of students who were (very) afraid of getting an infection was small (16%). This is in line with other results that have emphasized that for the young/students, the risk of the development of severe illness, symptoms, and complications is relatively low and therefore may not cause much concern [5, 38]. In one study, living in an urban area increased levels of anxiety [8]. In addition, it has to be noted that the federal state in Germany where the university of Lübeck is located is characterized by a more rural landscape, with only a few medium sized towns and a low population density. In terms of infection statistics of the COVID-19 pandemic, it was at the lower end of the German federal states [39]. Regarding mental health, we did not find significant differences in perceived stress and self-reported symptoms of depression and only a minor elevation for anxiety between 2019 and 2020 in the students who responded at both timepoints. This may sound contradictory to reports about high proportions of students with symptoms of anxiety and depression in cross-sectional studies [7–9, 12] and elevated stress, anxiety, loneliness, and depressive symptoms of Swiss and Italian students in longitudinal analyses [13, 14]. However, the analysis of study-related behavior and experience patterns (AVEM) revealed that the study participants were not uniform but divided into four distinct patterns. Here there was a significant difference between the total distribution in 2019 and 2020 and interesting developments. While the proportion of students with a healthy pattern and the risk pattern for overexertion slightly decreased (36 to 31% and 20 to 18%, respectively), the proportion of those with an unambitious pattern and a risk pattern for burnout increased (26 to 31% and 19 to 20%, respectively). The changes in behavior and experience patterns (AVEM) from 2019 to 2020 gives the impression of slightly subdued health and engagement. This fits with the perceived impact on study and intangible existence in our data and the reported perception of other than normal study mostly with digital/online based lectures and studying at home [40]. However, these perceptions may not be significantly detected by the standard items or instruments for general health, anxiety, or depression used in our study. Consistently, in regression analysis, the most important predictors for anxiety in 2020 were high scores in anxiety and depression in 2019. Gender and factors associated with the COVID-19 pandemic were less important.

### Preventive measures

The preventive measures of wearing a mask and washing hands more often related to the COVID-19 pandemic were followed at least sometimes by almost all of the students. This positive adoption of general recommendations from studies has been reported worldwide [2] but has not been seen in all settings. Considering pupils in Wuhan, China, lower proportions with appropriate behavior were reported [16]. Harper et al. [41] reported that the fear of a SARS-COV-2 infection was an important predictor for preventive measures. Although the proportion of students who were afraid of an infection was small in our sample, in regression analysis the fear of infection and the fear of consequences for health and economic existence indeed attributed significantly to the proportion of explained variance for washing hands more often.

Of interest was the high expression of more indirect measures led by contacting friends and family. Through social distancing, online teaching and learning at home, sheltering in place, or even quarantining, many students have expressed feelings of isolation and loneliness [11]. This could lead to mental health symptoms or unproductive coping mechanisms like substance abuse [42]. Social support, on the other hand is one of the most effective measures to prevent stress and mental health symptoms [20, 43, 44]. Social support by relatives decreased the risk for mental health symptoms in students during the COVID-19 pandemic [8]. Women tend to be more engaged in relationships especially in times of stress and also give more social support compared with men [45, 46]. In our study, a significantly higher proportion of female students contacted friends and family in response to the COVID-19 pandemic compared with their male fellow students (76% vs. 57%). Eating a balanced diet, getting enough sleep, exercising regularly, and taking more care about cleanliness followed as more indirect preventive measures with more than 50% (up to 67%) who did so always/often. Using disinfectants (39%) and especially taking herbal supplements (15%) were less often reported. Again, a significantly higher percentage of women in our study was engaged in these specific and unspecific preventive measures compared to their male fellow students (exercise only descriptively). The higher engagement of women in preventive measures has been frequently reported [47]. In university students, ambiguous results have been found. A recent review did not support gender as a moderator for sleep quality in university students [17]. By contrast, being female was the most frequent predictor for the consumption of vegetables [48]. From a study in four European countries, female students were less prone to fast food and consumed fruit and salads but also sweets and cakes more often than their fellow male students [49]. Preventive measures regarding infectious diseases have also been more frequently reported for women compared with men [50]. In eight of 14 studies, women washed hands significantly more frequently after the severe acute respiratory syndrome (SARS) outbreak in 2003 [51]. This has also been reported for the COVID-19 pandemic [52]. In Iranian medical students, women had higher scores of preventive measures during the COVID-19 pandemic [53].

Studies on health behavior of STEM students are rare. In one such study, natural sciences students had lower scores in life satisfaction and perceived health compared with sports or education students [54]. In the present study, in all indirect preventive measures, STEM students scored lower in the top two boxes (always/often) compared with MED and HSC students, except taking herbal supplements (however this was used by only one quarter of students). In another study of first year students of medicine, law, or teaching, medicine students also showed slightly better preventive behavior patterns [55].

### Risk distribution in different student groups

We saw the first differences in student groups regarding the perception of the impact that the COVID-19 pandemic had had on their study or their economic and intangible existence. Those who rated the impact as high had higher stress and mental health scores of anxiety and depression. Regarding intangible existence, it is notable that from other studies not only feelings of boredom were reported facing the COVID-19 pandemic [56, 57], but also feelings of emptiness and sadness about the loss of normal life, which can even lead to a loss of meaning in life [58].

It should be noted that stress, anxiety and depression in 2019 explained a greater part of the variance for anxiety in 2020 than factors related to the COVID-19 pandemic. Students with a preexisting condition for mental health symptoms may therefore be perceived as an important target group for health-promoting measures.

The vulnerability of distinct student groups was supported by the further analysis of the differences of behavior and experience patterns (AVEM). In particular, those students with the risk patterns for overexertion and burnout (AVEM) presented with behavior that adds to the impression of a health risk. As expected, the healthy and the unambitious pattern differed from both risk patterns with lower scores of anxiety and depression. For the latter, a significant difference was also found between the risk patterns themselves. Differences in mental health between risk patterns and healthy or unambitious patterns were also confirmed by findings in physicians [59] and teachers [23]. Students with risk patterns also had significantly higher scores for taking supplements than the healthy and the unambitious patterns. For the more specific preventive measures (wearing a mask, washing hands, more cleanliness, using disinfectants), the greatest and often significant differences were seen between the unambitious pattern S and the risk pattern for overexertion, while the scores for the healthy pattern and the risk pattern for burnout were intermediate. Even more interesting was the fact that for nutrition and exercise as well as for social support, those students with the healthy pattern showed the highest scores, and those with risk pattern for overexertion and burnout showed the lowest scores. In sum, these findings underline that the risk patterns correlate to mental health symptoms and illness. Students with these risk patterns tend to neglect measures of health promotion and to use seemingly easy but unproductive ways to cope (e.g., supplements).

### Recommendations for practice and future research

Although we did not find marked increases in mental health problems in these German students, the proportions with the perception of stress and symptoms of anxiety and depression as well as risk patterns of overexertion and burnout were relatively high. Measures, currently summarized under the heading “student health management” should be targeted and tailored for students at risk. A higher vulnerability of female students has to be taken into consideration. Measures should not only focus on students’ behavior (relaxation, sleep, exercise, etc.) but also address contextual factors (frequency and modes of testing and examination, workload and material to be learned, etc.) [60]. There are several public/political initiatives to address critical developments in students’ life’s due to the COVID-19 pandemic. For example, there are measures to improve digital teaching [61], to alleviate financial shortcomings [62], or to foster research projects for the evaluation of long-term effects of the COVID-19 pandemic [63, 64]. This also includes initiatives for maintaining and fostering mental health [65].

Because our survey was conducted during the early phase of the COVID-19 pandemic, follow-up surveys are necessary to detect possible later onset of mental health problems. In a survey at a German university, 13% of students admitted to consuming more alcohol since the COVID-19 pandemic had begun [11]. Research should therefore include the issue of substance-related addiction (e.g., alcohol, medication, marijuana) or substance-free addiction (e.g., gaming) as possible dysfunctional coping mechanisms.

### Strengths and limitations

A strength of our study is that we were able to analyze the impact of the COVID-19 pandemic in a prospective, longitudinal design. Response rates of students of all faculties were good. A limitation, however, may be that a relatively higher response rate of medical and female students does not allow us to rule out entirely selection biases. In addition, our data from one German university may not be representative for all German students.

## Conclusion

Comparison of the scores of psychosocial stress and mental health symptoms from 2019 and 2020 for the total study group did not support a strong effect of the COVID-19 pandemic in the students of this German university. However, more vulnerable student groups were identified (preexisting stress, anxiety, depression, overexertion, burnout). Measures of prevention and health promotion should be targeted especially at these groups. It is important to notice that in women a higher vulnerability is associated with more frequent practice of more productive coping measures.

## Supplementary Information

**Additional file 1: Supplementary Table S1.** Correlation matrix of included variables

**Additional file 2. **Questionnaire

## Data Availability

The datasets used and/or analyzed during the current study are available from the corresponding author on reasonable request.

## Abbreviations

AVEM: “Work-related behavior and experience pattern” [Arbeitsbezogenes Verhaltens- und Erlebensmuster]
HSC: Health sciences students
MED: Medicine students
STEM: Science, technology, engineering and mathematics students
