# Supplementary material for: The impact of the COVID-19 pandemic on stress, mental health and coping behavior in German University students – a longitudinal study before and after the onset of the pandemic
Source: BMC Public Health. 2021 Jul 13;21:1385. doi: 10.1186/s12889-021-11295-6 (PMC8275908; doi:10.1186/s12889-021-11295-6)
Supplement: Supplementary file 2 — Additional file 2. Questionnaire [file 12889_2021_11295_MOESM2_ESM.docx]

## Supplement - Questionnaire

**The Impact of the COVID-19 Pandemic on Stress, Mental Health and Coping Behavior in German University Students – A Longitudinal Study Before and After the Onset of the Pandemic**

Edgar Voltmer^a^, Susen Köslich-Strumann^a^, Anna Walther^a^, Mahmoud Kasem^a^, Katrin Obst^a^, Thomas Kötter^b^

#### General Health [1]

How would you describe your general health?

very good, good, satisfactory, poor, very poor

#### Selected items on COVID perceptions and measures

Did you have contact to a person infected with coronavirus?

(yes/no)

Did you yourself suffer a coronavirus infection?

yes, no, not known

How much are you afraid about a contagion with the coronavirus? [2]

very afraid, afraid, slightly afraid, almost not afraid

Supposed you were infected by COVID-19, how seriously do you think COVID-19 would affect your health?[3]

Extremely, very seriously, seriously, moderate, not much

Which areas of your life are severely affected by the corona crisis?

my studies: not at all, not much, neutral, somewhat, very much

economic existence: not at all, not much, neutral, somewhat, very much

intangible (emotion, meaning) existence: not at all, not much, neutral, somewhat, very much

How often do you use the following health precautions? [4]

wearing a mask: never, seldom, sometimes, often, always,

washing hands more often

taking more care about cleanliness

using disinfectants

eating a balanced diet

exercising regularly

taking an herbal supplement

making sure getting sufficient sleep

contact friends and family

#### Perceived Stress Scale (PSS) [5].

In the last month, how often …

…have you been upset because of something that happened unexpectedly?

never, almost never, sometimes, fairly often, very often (also for the following)

…have you felt that you were unable to control the important things in your life?

…have you felt nervous and “stressed”?

…have you felt confident about your ability to handle your personal problems?

…have you felt that things were going your way?

…have you found that you could not cope with all the things that you had to do?

…have you been able to control irritations in your life?

…you felt that you were on top of things?

…you been angered because of things that were outside your control?

…have you felt difficulties were piling up so high that you could not overcome them?

#### Brief Symptom Inventory (BSI-18) [6]

During the past 7 days, how much were you distressed by:

Faintness or dizziness: Not at all, a little bit, moderate, quite a bit, extremely (also for the following)

Pains in the heart or chest

Nausea or upset stomach

Trouble getting your breath

Numbness or tingling in parts of your body

Feeling weak in parts of your body

Feeling no interest in things

Feeling lonely

Feeling blue

Feelings of worthlessness

Feeling hopeless about the future

Thoughts of ending your life

Nervousness or shakiness inside

Feeling tense or keyed up

Suddenly scared for no reason

Spells of terror or panic

Feeling so restless you couldn’t sit still

Feeling fearful

#### Work-Related Behavior and Experience Pattern (AVEM) [7]

Studying is the most important element in my life.

I strongly agree, I somewhat agree, I`m in the middle, I somewhat disagree, I strongly disagree *(also for the following)*

As far as my career is concerned, I consider myself to be fairly ambitious.

If necessary, I will work until I am exhausted.

My work should never contain errors or deficiencies.

I still go on thinking about study issues in my leisure time.

I quickly resign myself to lack of success.

Lack of success can challenge me to try harder.

I don’t get upset easily.

Until now I have been successful in school/my studies.

I have good reason to look into the future with optimism.

My partner (or the person to whom you are closest) shows understanding for my studies.

My studies are everything to me.

In terms of career, I am aiming to get further than most other people.

I always give it all I’ve got.

I prefer to check everything three times over rather than hand in work that contains mistakes.

After lectures I can switch off easily.

I find it difficult to cope with lack of success.

If I don´t succeed in something, I don´t give up, but try even harder.

I consider myself to be rather hectic.

Up to this point in my career, I have experienced more success than disappointments.

I have no reason at all to be dissatisfied with my life.

My family isn’t very interested in my problems regarding my studies.

I need the work on study issues like the air I breathe.

I have great plans for my studies/my future career.

I work more than I really should.

Whatever I do, it must be perfect.

Problems with my studies occupy my mind all day.

Failure in my studies is very discouraging for me.

Lack of success doesn’t discourage me, but makes me try even harder next time.

I remain calm in the midst of turmoil.

So far, I have been very successful in my development.

So far, I have been satisfied with my life.

I would like my partner (or the person to whom you are closest) to have more consideration for my study-related tasks and problems.

I don´t know what I should do other than studying.

Success at work is an important aim in my life.

I tend to overwork.

I don´t consider my work to be finished until I am completely satisfied with the result

My thoughts are always circling around my studies.

Failure in my studies makes me very depressed.

If I don´t succeed, I say to myself: “This time nothing will stop me!”.

I can remain calm and collected in almost all situations.

My life up till now has been characterized by success.

By and large, I am happy and content.

I have the full support of my family.

### References

1. RKI. Daten und Fakten: Ergebnisse der Studie »Gesundheit in Deutschland aktuell 2012. Berlin: Robert-Koch Institut; 2014.

2. Statista (2020) Wie groß ist Ihre Angst vor einer Ansteckung mit dem Coronavirus (COVID-19)? . https://de.statista.com/statistik/daten/studie/1096524/umfrage/umfrage-zur-angst-vor-ansteckung-mit-dem-coronavirus-in-deutschland/.

3. IPSOS (2020) COVID-19-Pandemie: Mehrheit der Deutschen bezweifelt,dass sich die Ausbreitung des Virus stoppen lässt. https://www.ipsos.com/sites/default/files/ct/news/documents/2020-03/ipsos-pi_coronatracker_wave5_mar2020.pdf.

4. Lee-Baggley D, DeLongis A, Voorhoeave P, Greenglass E. Coping with the threat of severe acute respiratory syndrome: Role of threat appraisals and coping responses in health behaviors. Asian journal of social psychology. 2004; 7(1):9-23. 10.1111/j.1467-839X.2004.00131.x.

5. Cohen S, Kamarck T, Mermelstein R. A global measure of perceived stress. J Health Soc Behav. 1983; 2410.2307/2136404.

6. Franke GH, Jaeger S, Glaesmer H, Barkmann C, Petrowski K, Braehler E. Psychometric analysis of the brief symptom inventory 18 (BSI-18) in a representative German sample. BMC medical research methodology. 2017; 17(1):14. 10.1186/s12874-016-0283-3.

7. Schaarschmidt U, Fischer AW. Arbeitsbezogenes Verhaltens- und Erlebensmuster AVEM, 3rd edn. Frankfurt a. M.: Swets & Zeitlinger; 2008.
